# Ezrin expression in female reproductive tissues: A review of regulation and pathophysiological implications

**DOI:** 10.3389/fcell.2023.1125881

**Published:** 2023-03-08

**Authors:** Wen-Ting Xu, Ling-Li Shi, Jie Xu, Haiqing Qian, Huifang Zhou, Li-Hong Wang

**Affiliations:** ^1^ Department of Reproduction, Zhangjiagang TCM Hospital Affiliated to Nanjing University of Chinese Medicine, Zhangjiagang, Suzhou, Jiangsu, China; ^2^ Translational Medical Innovation Center, Zhangjiagang TCM Hospital Affiliated to Nanjing University of Chinese Medicine, Zhangjiagang, Suzhou, Jiangsu, China; ^3^ Department of Gynaecology, Affiliated Hospital of Nanjing University of Chinese Medicine, Nanjing, China

**Keywords:** ezrin, female reproductive, embryo, oocytes, follicle

## Abstract

Ezrin, a plasma membrane-microfilament linker, is a cytoskeletal organizer involved in many cellular activities by binding to the membrane protein-ezrin-cytoskeletal protein complex and regulating downstream signal transduction. Increasing evidence demonstrates that ezrin plays an important role in regulating cell polarity, proliferation and invasion. In this study, we analyzed the effects of ezrin on oocytes, follicle development, embryo development and embryo implantation. We reviewed the recent studies on the modalities of ezrin regulation and its involvement in the biological processes of female reproductive physiology and summarized the current research advances in ezrin inhibitors. These studies will provide new strategies and insights for the treatment of diseases.

## 1 Introduction

Ezrin, also known as cytovillin and violin-1, was one of the first important members of the ezrin-radixin-moesin (ERM) family to be discovered. 1981, Hunter et al. discovered rapid phosphorylation of tyrosine residue of an approximately 81 kD polypeptide, the first time that ezrin was discovered as a substrate for the epidermal growth factor receptor protein tyrosine kinase. In 1983, an 80 kD protein was purified during identifying the microvilli fraction and named ezrin after the university (Ezra Cornell) (Barik et al., 2022).

**FIGURE 1:**
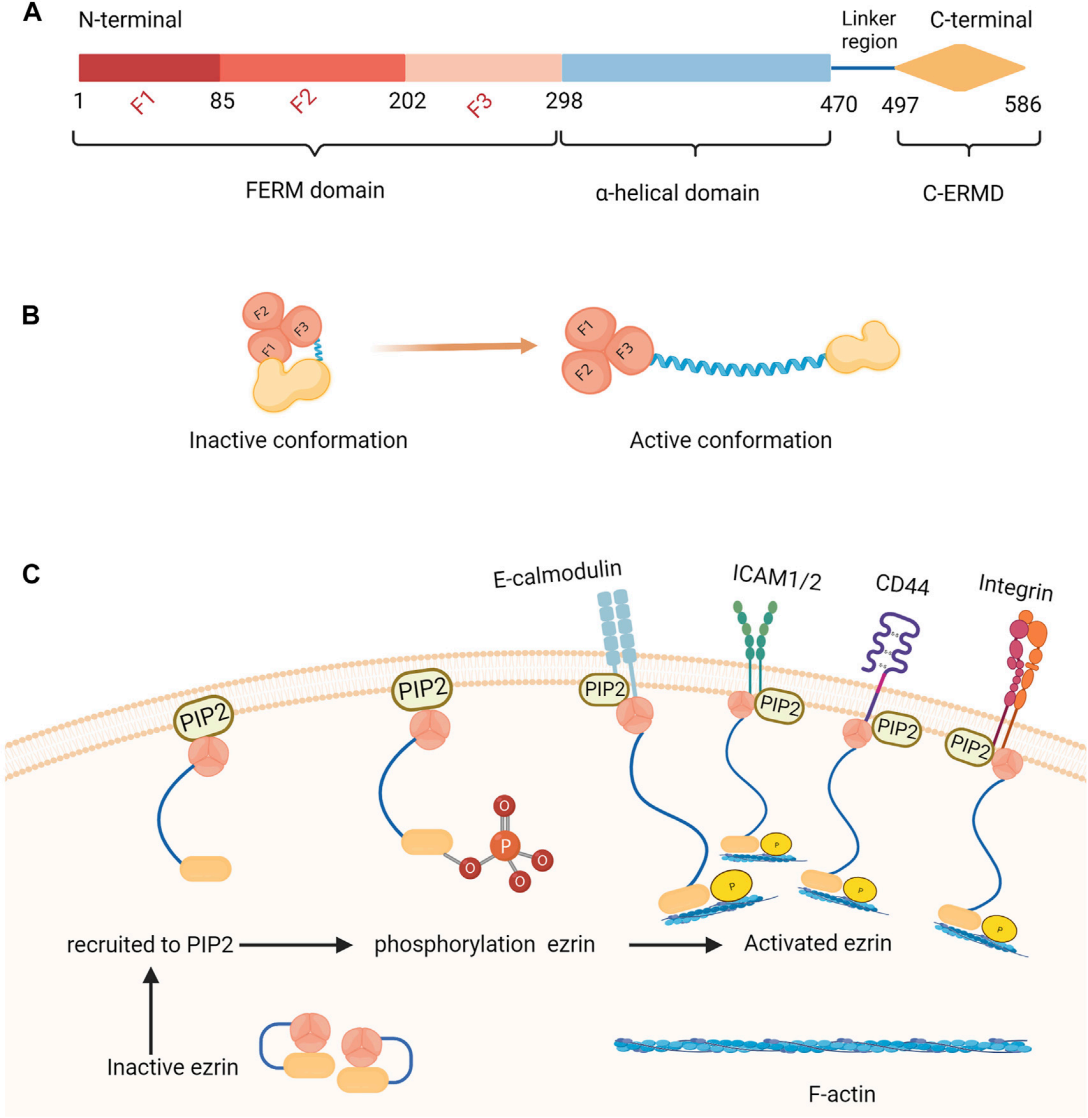
Structure and activation process of ezrin. **(A)** Ezrin is composed of 586 amino acids and contains three main structural domains: The domain structure includes the FERM domain (N-terminal) which is a highly conserved globular structure of 296 amino acids and can be directly or indirectly attached to the cell membrane, the α-helix structural domain and the C-ERMAD domain (C-terminus) which can connect to F-actin and the FERM region. **(B)** The inactive state and active conformation of ezrin:Ezrin is inactive in the resting state, but it connects to the cell membrane and cytoskeleton when phosphorylated and reverts to a closed state after dephosphorylation. **(C)** The process of the activation of ezrin: Ezrin can become activated by phosphorylation of the C-terminal threonine residue (Thr567), phosphorylation of tyrosine (Tyr353) or N-terminal binding to phosphatidylinositol biphosphate (PIP2). Activated ezrin protein mediates signaling pathways though bonding to the molecules on the cell membrane surface to maintain apical-basal cell polarity and normal cell morphology, and remains the consistency of cell-cell contacts through binding to actin filaments. Ezrin protein also participates in cell adhesion and can form complexes on the cell surface with various cell adhesion molecules such as CD44, E-calmodulin, and integrin, which are involved in cell adhesion, deformation, and migration by regulating actin shape.

Ezrin is composed of 586 amino acids and contains three main structural domains: 1. the amino-terminal (N-terminal) is a highly conserved globular structure of 296 amino acids called the FERM structural domain, which can be directly or indirectly attached to the cell membrane and can bind to CD44, CD43, ICAM-1, ICAM-2, ICAM-3, c-Met, E-Cadherin and other membrane proteins; 2. the middle is the α-helix structural domain; 3. the carboxyl terminus (C-terminus) is the positively charged actin-binding region, which can connect to F-actin and the FERM region (Figure 1A) (Kawaguchi and Asano, 2022). Ezrin is mainly distributed under the microvilli, membrane folds, pseudopods and other plasma membranes with special morphology. It is the cytoskeleton organizer and acts as a “scaffold” by forming the membrane protein-ezrin-cytoskeleton protein complex, which is involved in many cellular activities and regulates biological processes. Ezrin is inactive in the resting state, but it connects to the cell membrane and cytoskeleton when phosphorylated and reverts to a closed state after dephosphorylation. Ezrin has multiple binding sites that can be phosphorylated and can be converted from a dormant to an activated state by changing its conformation. Ezrin can become activated by phosphorylation of the C-terminal threonine residue (Thr567) (Xue et al., 2020), phosphorylation of tyrosine (Tyr353) (Liu et al., 2018) or N-terminal binding to phosphatidylinositol biphosphate (PIP2) (Ramalho et al., 2020). Activated ezrin protein mediates signaling pathways though bonding to the molecules on the cell membrane surface to maintain apical-basal cell polarity and normal cell morphology, and remains the consistency of cell-cell contacts through binding to actin filaments (Song et al., 2020a). Ezrin protein also participates in cell adhesion and can form complexes on the cell surface with various cell adhesion molecules such as CD44, E-calmodulin, and integrin, which are involved in cell adhesion, deformation, and migration by regulating actin shape (Figures 1B, C) (Distel et al., 2022). Ezrin has been extensively studied in the field of tumorigenesis, with research focusing on its role in regulating cell migration, proliferation, differentiation, and apoptosis, as well as mediating cell-cell signaling to control tumor growth, differentiation, and apoptosis (Barik et al., 2022). In some biological processes of female reproduction, such as endometrial hyperplasia and embryo implantation, tumor-like biological properties exist (Li et al., 2022). Recent studies have shown that the expression and localization of ezrin have been altered in tissues related to endometriosis (Peloggia et al., 2022), recurrent miscarriage (Zhang et al., 2022a), gestational trophoblastic disease (Tameishi et al., 2021), and polycystic ovary syndrome (PCOS) (Demacopulo and Kreimann, 2019), sparking our interest. This review provides a summary of recent studies on the modalities of ezrin regulation and its involvement in the biological processes of female reproductive system physiopathology.

## 2 The biological function of ezrin in the female reproductive system

### 2.1 Ezrin is involved in the arrangement of cytoskeleton in endometrium

In the endometrium, ezrin is mainly found around the perisecretory vesicles, in the parietal membrane of glandular epithelial cells and the cytosolic protrusions of microvillous epithelial cells. The endometrium progresses through three stages during a normal menstrual cycle: a proliferative phase, a transition to a secretory phase, and either decidualization or tissue degradation, depending on the presence of an embryo. The periodic alteration of the endometrium is vital for initiating and sustaining a successful pregnancy. Cellular and tissue remodeling is necessary for all these activities, and ezrin is needed to rearrange the cytoskeleton and cell-cell attachment in the endometrium (Peloggia et al., 2022). It was observed that the expression of ezrin was significantly enhanced during the secretory phase, and during the implantation window, ezrin was detected to enter the glandular lumens (Guo et al., 2021). Pinopode is an indicator of endometrial receptivity (Quinn et al., 2020). Immunohistochemistry has revealed the presence of ezrin in pinopode-laden uterine luminal epithelial cells. Ezrin can interact with thrombomodulin (TM), a membrane protein critical for the formation of the placenta. Ezrin-Thrombomodulin, a complex protein, facilitates the connection of actin filaments of the cytoskeleton to the cell surface, thus enabling the expression and arrangement of pinopodes (Peloggia et al., 2022). During implantation, estrogens and progesterone trigger the binding of TM to ezrin, as well as the phosphorylation and activation of ezrin. Activated ezrin then crosslinks F-actin into an orthogonal meshwork, concentrates on the apical pole of cells and participates in the meshwork seen in the pinopodes (D'Ippolito et al., 2020).

### 2.2 Ezrin contributes to the development of oocytes and follicles

The growth of the microvilli membrane of the oocyte is fundamental for maintaining the structural stability of the oocyte (Czukiewska and Chuva de Sousa Lopes, 2022). Oocyte microvilli are constructed from a core of actin filaments and cytoskeletal proteins, such as ezrin. Ezrin protein interacts with protein kinase and links F-actin filaments, creating a scaffold-like structure that serves as the basis for the formation of microvilli. The ezrin–actin scaffold depends on the availability of EBP50, an ezrin-binding protein susceptible to the cAMP-dependent protein kinase (Kawaguchi and Asano, 2022). EBP50 links ezrin to membrane, thus enabling the completion of microvilli formation by attaching ezrin–actin.

The meiosis of the oocyte is necessary for its development and maturation. As mammalian oocytes mature, their cortical structure also undergoes alterations. Ezrin protein has been shown to promote meiosis by regulating the stiffness of the cell cortex, modulating the mechanical properties of the oocyte, and regulating the polarity of the oocyte. Mouse oocyte tension drops approximately six-fold from phase I to phase II during meiotic maturation and then increases 1.6-fold at fertilization (Özgüç et al., 2022). During oogenesis, active pERMs (Phospho-Ezrin/Radixin/Moesin) are localized in the oocyte cortical and the expression decreases during meiotic maturation to MII. However, after fertilization, their presence increases again, reflecting the dynamic tension changes that occur during these developmental stages (Zhuan et al., 2022).

### 2.3 Ezrin induces polarization of embryo

The polarization of the embryo is crucial for its development. In the early stages of mouse embryogenesis, epithelial cells undergo cell polarization, cell morphological changes, tissue type plasticity, and cell migration (Thowfeequ et al., 2022). During the development of the mouse embryo prior to implantation, the spatial redistribution of E-calmodulin induces a remodeling of the cytoskeletal structure and increases the intercellular contact area, contributing to the first sign of polarization - the formation of the apical ezrin-rich microvilli structural domain (Płusa and Piliszek, 2020). It was observed that before the 8-cell embryo polarization, ezrin was uniformly distributed over the entire cell surface. Once ezrin was phosphorylated, it aggregated from the cell junctions to the apical region, causing the microvilli to also aggregate in the apical region, indicating that phosphorylation of ezrin threonine-567 is necessary for the redistribution of embryo polarization (Yildirim et al., 2021; Zhang et al., 2022b). Recent studies have revealed that transcription factor AP-2 gamma (Tfap2c) and TEA domain transcription factor 4 (Tead4) can recruit ezrin proteins to localize to the membrane and promote the polarized growth of apical protein clusters, which eventually leads to apical protein centration. Tfap2c and Tead4 control the positive feedback-like synergistic recruitment of ezrin, while RhoA promotes membrane mobility, leading together to the formation of the apical region and the onset of polarization (Zhu et al., 2020).

### 2.4 Ezrin promotes trophoblast cell fusion and proliferation

The cAMP–PKA signaling pathway is involved in the process of trophoblast fusion. Ezrin was shown to direct protein kinase A (PKA) to a molecular complex of connexin 43 and zona occludens-1 through immunoprecipitation and immunolocalization experiments. It plays a role in promoting gap junctional communication by facilitating the phosphorylation of Cx43 (phosphorylation of S369 and S373) by PKA. This phosphorylation allows for the proper assembly and function of gap junctions, which leads to cell fusion in cytotrophoblasts (Dukic et al., 2018).

Research has revealed that the expression of CDC42 is significantly decreased in the villi tissue of recurrent spontaneous abortion (RSA) patients. The human trophoblast stem cells (hTSCs) model revealed a key role of CDC42 in controlling stemness and proliferation in hTSCs, which is mediated by ezrin to activate YAP in the nucleus (Shilei et al., 2022).

### 2.5 Ezrin participates in cell migration and invasion

Ezrin is a protein that is necessary for cell migration and invasion. Elevated expression of ezrin was observed in ectopic endometrium, which is consistent with the clinical stages of endometriosis. To evaluate the role of ezrin phosphorylation in cellular activities such as migration and invasion, cells were treated with an inhibitor of ezrin T567. The migration rate of ESCs treated with inhibitor was observed to be lower than the control group and the invasion of the inhibitor group was found to be decreased compared to the control group. It indicated that ezrin T567 phosphorylation is responsible for regulating the migration and invasion of ectopic ESCs(Chen et al., 2020).

Proliferation, invasion, adhesion are all essential biological processes for successful embryo implantation. The successful migration and penetration of extravillous trophoblast cells into the maternal decidua and myometrium is a major factor for the proper development of the embryo. During the implantation of embryos, trophoblasts exhibited some tumor-like behaviors, such as epithelial-to-mesenchymal transition and reorganization of the actin cytoskeleton, in order to enable dynamic cell elongation and directional motility (Li et al., 2022). It has been reported that inadequate trophoblast cell invasion is a vital factor in various pregnancy-related disease, such as RSA. *In vitro* experiments confirmed that knockdown of ezrin expression significantly inhibited trophoblast cell invasiveness. The trophoblast cells were incubated with PKC inhibitor Gö6976 and activator TPA, and it was found that Gö6976 significantly reduced the invasive ability of trophoblast cells. At the same time, TPA increased the relative invasive ability of trophoblast cells. Moreover, p-ezrin levels were significantly decreased in Gö6976-treated trophoblast cells, and increased expression of phospho-ezrin was observed in TPA-treated trophoblast cells, in accordance with the expression of phosphorylated PKC. The results suggest that PKC may induce trophoblast cell infiltration by activating ezrin and that ezrin may promote embryonic attachment by regulating the invasiveness of trophoblast cells (Zhang et al., 2022a).

## 3 The post-translational modifications of ezrin

### 3.1 Three main post-translational modifications of ezrin

Post-translational modifications, including phosphorylation, ubiquitination and acetylation, have been observed to regulate the cellular function of Ezrin.

**FIGURE 2:**
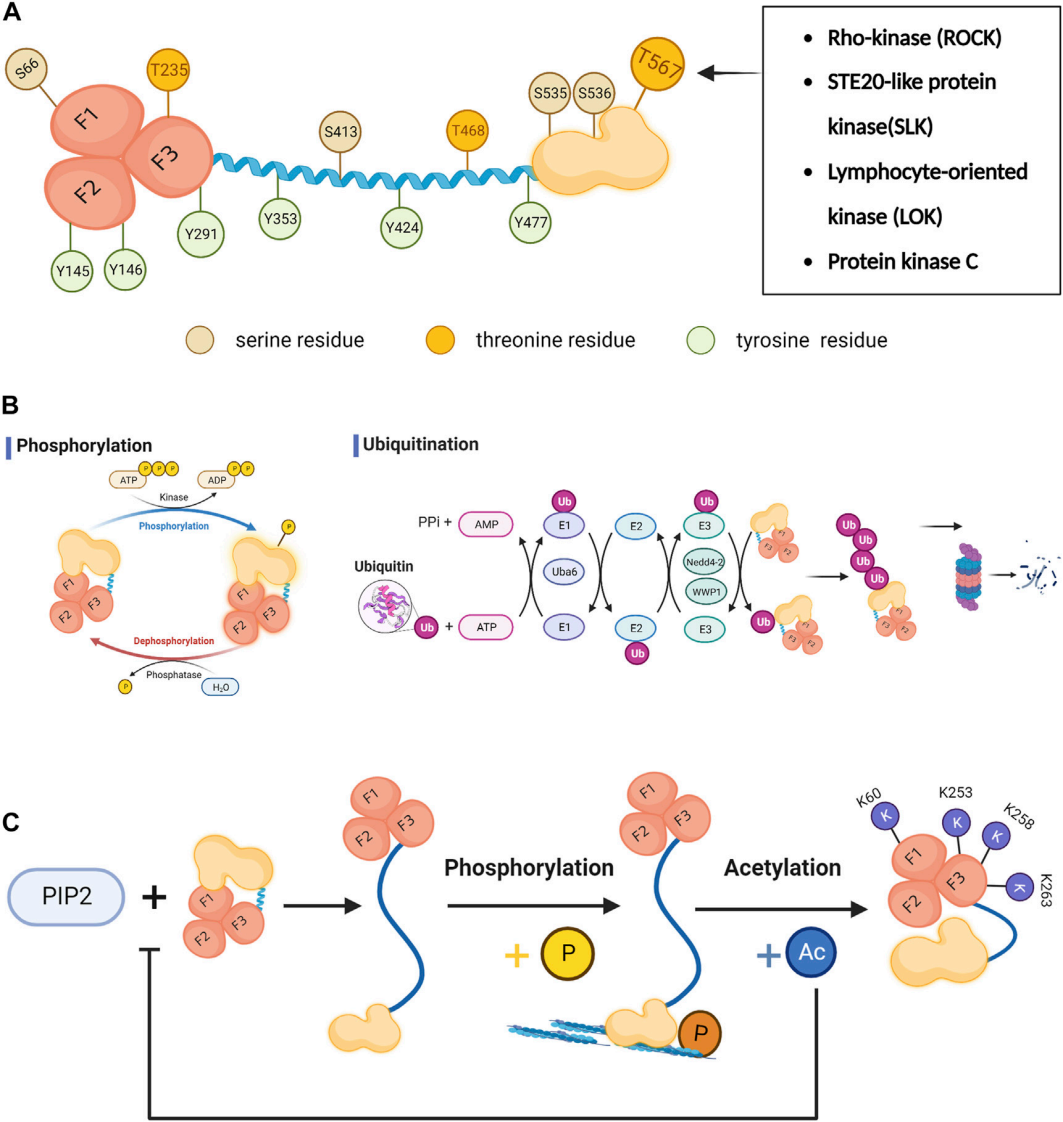
The post-translational modifications of ezrin. **(A)** The active state of ezrin protein and the phosphorylation sites:T567, S535 and S536 are located at the C-ERMAD terminus, of which T567 is the most studied phosphorylation site; Y477 is located in the Linker region; Y424, Y353, S413, T468 exist in the α-helical domain; N-ERMAD terminus contains S66(F1), Y145(F2), Y146(F2), T235(F3), Y291(F3). Among them, T235, T468 and T567 are threonine residues. S66,S413, S535 and S536 are serine residues. Y145, Y146, Y291,Y353, Y424 and Y477 are tyrosine residues. The critical T567 on C-ERMAD could be phosphorylated by Rho-kinase (ROCK), STE20-like protein kinase (SLK), lymphocyte-oriented kinase (LOK)and protein kinase C(PKC). **(B)** The phosphorylation and ubiquitination process of ezrin:Protein ubiquitination is mediated sequentially by ubiquitin-activating enzyme E1, ubiquitin-binding enzyme E2, and ubiquitin-ligase E3. The ubiquitin-activating enzyme E1 includes UBA1 and UBA6. **(C)** Acetylated lysine sites exist in the FERM structural domain of ezrin (K60, K253, K258 and K263). Ezrin acetylation prevents Thr567 phosphorylation. Acetylated ezrin can lead to the translocation of ezrin from the cell membrane to the cytoplasm by affecting the affinity of its C-ERAM to F-actin, reducing the binding of the N-FERM structural domain to PIP2, membrane-associated proteins and cell adhesion molecule.

Phosphorylation is currently the most well-studied post-translational modification of ezrin (Michie et al., 2019). Among the reported phosphorylation sites, T567, S535 and S536 are located at the C-ERMAD terminus, of which T567 is the most studied phosphorylation site (Xue et al., 2020); Y477 is located in the Linker region; Y424, Y353, S413, T468 exist in the α-helical domain; N-ERMAD terminus contains S66(F1), Y145(F2), Y146(F2), T235(F3), Y291(F3) (Figure 2A) (Michie et al., 2019; García-Ortiz and Serrador, 2020; Barik et al., 2022).

Protein ubiquitination is mediated sequentially by ubiquitin-activating enzyme E1, ubiquitin-binding enzyme E2, and ubiquitin-ligase E3. The human genome contains two ubiquitin-activating enzymes E1, Uba1 and Uba6. The Uba1 protein has been thought to be the only one that plays a role in all ubiquitination reactions E1. until it was confirmed that it is Uba6 that plays an important role in the polyubiquitination of ezrin. Silencing of UBA6 leads to reduced polyubiquitination of ezrin and it is involved in the localization of ezrin within MCF-10A cells and the control of epithelial morphogenesis (Figure 2B) (Liu et al., 2017).

In addition, acetylation is also a post-translational modification of ezrin. Ezrin acetylation, unlike phosphorylation, negatively regulates ezrin and cross-talks with the phosphorylation of the T567 site. Ezrin acetylation prevents Thr567 phosphorylation, enhances the association between the N-terminal and C-terminal domains within the molecule, and converts ezrin to an inactive reactive concept. There are several potential acetylated lysine sites in the FERM structural domain of ezrin (K60, K253, K258 and K263). Acetylated ezrin can lead to the translocation of ezrin from the cell membrane to the cytoplasm by affecting the affinity of its C-ERAM to F-actin (Song et al., 2020b), reducing the binding of the N-FERM structural domain to PIP2,membrane-associated proteins and cell adhesion molecule (Figure 2C) (Rahimi et al., 2021).

### 3.2 Post-translational modifications of ezrin in female reproductive disorders

In female reproductive endocrine disorders, ezrin phosphorylation is the currently reported post-translational modification. Ezrin phosphorylation sites can be regulated by several signaling pathways and perform different physiological functions upon activation and phosphorylation (Yin and Schnoor, 2022). Initial studies demonstrated that the critical T567 on C-ERMAD could be phosphorylated by Rho-kinase (ROCK) (Ding et al., 2022), STE20-like protein kinase (SLK) (Garland et al., 2021), lymphocyte-oriented kinase (LOK) (Senju and Tsai, 2022) and protein kinase C(PKC) (Khan et al., 2022) both in the laboratory and in living organisms. Ezrin Y145, Y353 and Y477 can be modified by phosphorylation through the action of Src kinases and the intrinsic Tyr kinase activity of receptors for epidermal growth factor, hepatocyte growth factor and Platelet-derived growth factor (Huang et al., 2018; Derouiche and Geiger, 2019; Rainey et al., 2020).

The study on endometriosis suggested that Ezrin T567 phosphorylation has been involved in the remodeling of the cytoskeleton of ectopic endometrial stromal cells (ESCs) (Chen et al., 2020). It was observed that the levels of ezrin and Rho pathway were higher in ectopic endometrium, which is consistent with the clinical stages of endometriosis. The inhibition of ezrin phosphorylation (T567) resulted in a decrease in the expression of the Rho pathway. Therefore, we can infer that Rho pathway may be involved in the mechanism by which ezrin phosphorylation regulates cell migration and invasion *via* actin polymerization. In the study on the unexplained RSA (URSA) patients, the expression of ezrin and phosphorylated ezrin (T567) both decreased in the trophoblast cells in URSA patients in comparison to normal pregnant women. Furthermore, it demonstrated that suppressing ezrin expression could significantly reduce the invasiveness of trophoblast cells. In order to ascertain the kinase candidates responsible for the activation of Ezrin at T567, trophoblast cells were exposed to a PKC inhibitor. The results indicated a substantial decrease in the p-ezrin level of the treated cells, suggesting that PKC was the kinase responsible for the phosphorylation of ezrin (Zhang et al., 2022a). Furthermore, endometrial biopsy samples from RPL patients showed a decreased presence of both the phosphorylated (Y353) and total levels of ezrin in Western immunoblot analysis compared to the control group (D'Ippolito et al., 2020).

Placental dysplasia can lead to a heightened risk of recurrent spontaneous abortion. *In vivo* experiments indicated that phosphorylation of ezrin on T567 was responsible for the stemness and proliferation of human trophoblast stem cells (hTSCs). This phosphorylation further triggered the nuclear activity of Yeast Aspartyl Protease (YAP), thus suppressing the differentiation of hTSCs. It was observed that the expression of cyclin D1 and β-hCG in human trophoblast stem cells that had been depleted of CDC42 could be rescued by the overexpression of ezrin-T567D, an active form of ezrin that regulates membrane tension. All theses evidence suggests that CDC42/ezrin (T567) signaling is essential for YAP to be transported to the nucleus during the initial phases of placental formation (Shilei et al., 2022). Taken together, Studies of phosphorylation sites may offer a novel therapeutic target for diseases.

## 4 The post-transcriptional regulation of ezrin

Non-coding RNAs (ncRNAs), especially microRNAs (miRNAs) and long-stranded non-coding RNAs (lncRNAs), have recently attracted growing attention due to their multifunctional roles as key regulators of gene expression. Studies have confirmed that an increasing number of microRNAs and long non-coding RNAs are involved in the post-transcriptional regulation of ezrin, and cyclic RNAs have also been reported to play biological roles by binding to ezrin. The major microRNAs of ezrin include: miR-183 (Zhang and Wang, 2019; Cao et al., 2020), miR-200b (Yuan et al., 2020), miR-96 (Yao et al., 2018; Mao et al., 2019), miR-211 (Pei et al., 2019; Naso et al., 2020), miR-148b (Sun et al., 2018), miR-205-3p (Qiu et al., 2019), miR-25-3p (Rao et al., 2020), miR-335-5p (Tamanini et al., 2021), miR-462-731 (He et al., 2022), miR-802 (Ge et al., 2022), miR-204-5p (Jiang et al., 2021); the long non-coding RNAs (lncRNAs) and cirRNAs include: lncRNA EZR-AS1 (Liu et al., 2019; You et al., 2020; Lu et al., 2021), lncRNA KCNQ1OT1 (Zhang et al., 2018a), lncRNA TUG1 (Yao et al., 2022), circARHGAP12 (Fan et al., 2021) and circCDYL2 (Li et al., 2021) (Table 1).

**TABLE 1:** The post-transcriptional regulation of ezrin.

Non-coding RNA	Mechanism of action	Cell line	Pathways	Refs
circARHGAP12	promote NPC cell invasion and metastasis	NPC cell lines	TPM3/RhoA	Fan et al. (2021)
circCDYL2	promote CRC migration	CRC cell lines	Ezrin/AKT	Li et al. (2021)
LncRNA EZR-AS1	repress proliferation, migration and invasion of cSCC	CSCC cell lines	PI3K/AKT	Lu et al. (2021)
	regulate the proliferation, migration, and apoptosis of cells	HUVECs	SMYD3	You et al. (2020)
	Inhibit proliferation, invasion and migration of cells	human CRC cell lines	TGF-β	Liu et al. (2019)
LncRNA KCNQ1OT1	regulates proliferation and cisplatin resistance	CAL27 and SCC9 cell lines	Fak/Src	Zhang et al. (2018a)
LncRNA TUG1	promotes cell proliferation and invasion	SW1353 cells and hFOB1.19 cells	miR-377-3p	Yao et al. (2022)
miR-148b	increase resistance to CHOP in diffuse large Bcell lymphoma cells	DLBCL cell line CRL2631	N/A	Sun et al. (2018)
miR-183	inhibit A375 cell migration and invasion	A375 cell line	MMP-9	Zhang and Wang (2019)
miR-183-5p	inhibit the epithelial-mesenchymal transition, proliferation, invasion and migration	Ishikawa, KLE, JEc, HEc-1-A, and HHUA cells	E-cadherin	Yan et al. (2018)
miR-205-3p	promote the proliferation and invasion and reduce the apoptosis of breast cancer cells	Human breast cancer MCF-7 cell lines	N/A	Qiu et al. (2019)
miR-211	regulate cell proliferation, apoptosis and migration/invasion in human osteosarcoma	Human osteosarcoma cell line 143B	N/A	Pei et al. (2019)
miR-462-731	induce macrophage polarization to the M1 phenotype	C. idella kidney cells	ell1	He et al. (2022)
miR-802	increase F-actin rearrangement	Acinar cells	RhoA	Ge et al. (2022)
miR-96-5p	disrupte arrangement of cytoskeletons and impaire cell invasion ability	trophoblastic cells	cytoskeletons	Mao et al. (2019)
miRNA-204-5p	act as a tumor suppressor to influence astrocytoma invasion and migration by targeting ezrin	U87MG and LN382 cells	N/A	Jiang et al. (2021)

### 4.1 The regulation of ezrin by microRNAs

The luciferase reporter gene assay confirmed that miR-183 and miR-96-5p directly regulate ezrin. In the endometrial Ishikawa cell line, miR-183-5p targeted and negatively regulated ezrin expression. It was found to reduce ezrin expression to inhibit epithelial-mesenchymal transition (EMT), cell proliferation, migration and invasion; however, promoting apoptosis (Yan et al., 2018). Meanwhile, miR-183 inhibited embryo implantation by binding Heparin Binding EGF-like growth factor (HB-EGF) (Cao et al., 2020). Human trophoblast-derived cell, HTR8-SVneo, transfected with miR-96-5p showed significantly lower fluorescence intensity in pGL3-ezrin-miR-96-Mut compared to pGL3-ezrin-miR-96-WT group and negative control group. miR-96-5p can directly bind to EZR mRNA and inhibits translation of EZR mRNA to ezrin protein instead of enabling mRNA degradation. miR-96-5p downregulates ezrin protein levels, disrupting the cytoskeleton and impairing cellular infiltration ability. With the downregulation of miR-96-5p expression, the level of ezrin protein expression is also reversed, and cellular infiltration capacity is partially restored (Mao et al., 2019).

### 4.2 The regulation of ezrin by LncRNAs and cirRNAs

Luciferase assays demonstrated that miR-211-5p was able to bind to the 3′-UTR of ezrin mRNA, and its presence inhibited the proliferation of TSCC cells by targeting the Ezrin/Fak/Src signaling pathway. On the other hand, lncRNA KCNQ1OT1 was found to be effective in sponging miR-211-5p, thus promoting the progression of TSCC through the Ezrin/Fak/Src signaling pathway (Zhang et al., 2018a). Moreover, it was found that EZR-AS1 could stimulate cell migration by enhancing the expression of EZR. By forming a complex with RNA polymerase II, the antisense lncRNA EZR-AS1 is able to induce the transcription of EZR. EZR-AS1 facilitates the recruitment of SMYD3 to the SMYD3 binding site located in the GC-rich region downstream of the EZR promoter, which leads to an increased concentration of SMYD3 in the area and the trimethylation of H3K4 in the EZR gene. The collaboration between EZR-AS1 and SMYD3 amplifies the transcription and expression of EZR, thus stimulating the migration of ESC cells (Zhang et al., 2018b). circCDYL2 binds to ezrin mRNA and upregulates its protein levels, which can stabilize ezrin and protect it from ubiquitinated proteasomal degradation (Li et al., 2021). Analysis of sequencing data indicated the presence of a novel circRNA, circARHGAP12, with m (6)A modification, which was observed to be increased in cervical cancer tissue and cells (Ji et al., 2021), which was confirmed to bind directly to the 3′UTR of ezrin mRNA, promoting its stability (Fan et al., 2021). Although little research has been done on the regulation of ezrin by LncRNAs and cirRNAs in reproductive endocrine disorders, it is believed that potential links will emerge with further research in the post-transcriptional regulation of ezrin.

## 5 The pharmacological inhibition of ezrin

NSC305787 and NSC668394 are two inhibitors of ezrin that inhibit ezrin (Thr567) phosphorylation caused by PKCΙ primarily *via* their binding to ezrin (Qureshi-Baig et al., 2020). Studies are currently being conducted to investigate the antitumor effects of these two compounds, particularly on osteosarcoma (Shoaib et al., 2022), rhabdomyosarcoma (Proudfit et al., 2020), pancreatic cancer (Lipreri da Silva et al., 2022), and colon cancer (Qureshi-Baig et al., 2020). *In vitro*, cell culture and interference also confirmed that NSC305787 reduced the cellular and malignant molecular behavior of CLL cell lines (Lipreri da Silva et al., 2021).

Recently, it was found that ezrin and Rho pathway expression was higher in ectopic endometrium, and NSC305787 inhibited the phosphorylation of T567 ezrin, leading to a decrease in Rho pathway expression and a decrease in filamentous pseudopods in ectopic endometrial stromal cells. Primary cultured Endometrial stromal cells were exposed to 2.5 μM NSC305787 for 3 h. It was observed that the NSC305787-treated cells were more rounded with fewer filamentous pseudopods and scattered actin filaments. Western blot analysis revealed that the expression of p-ezrin and RhoA/RhoC/ROCK1 was significantly reduced in cells that were treated with both 2.5 and 5 μM NSC305787 compared to the control cells (Chen et al., 2020). This provides a potential target for the treatment of endometriosis.

## 6 Conclusion

As a mediator between the plasma membrane and the actin cytoskeleton, ezrin plays a key role in maintaining cell structure, forming specific cellular structures, maintaining cell morphology, and regulating cell motility functions. Ezrin can regulate downstream signaling pathways by binding to cell adhesion molecules on the cell surface, for instance, ezrin can regulate follicular development through the PKC signaling pathway and increase the invasive capacity of trophoblast cells. It has been shown that ezrin also regulates the reorganization of the actin skeleton, so as to modulate endometrial receptivity and promote the adhesion of blastocysts to endometrial epithelial cells. All these results suggest that ezrin plays an important role in the pathological development of gynecological diseases, such as PCOS, endometriosis, recurrent miscarriage, endometrial cancer and other gynecological tumors. Meanwhile, with the in-depth study of ezrin inhibitors, it will provide us with new insight and orientation for the treatment of diseases.
